# Non-Oxidised Parathyroid Hormone and a Panel of Markers of Calcium–Phosphate Metabolism for Analysis of Secondary Hyperparathyroidism in Selected Patient Groups—A Quality Assurance Project

**DOI:** 10.3390/ijms26094279

**Published:** 2025-04-30

**Authors:** Ursula Huber-Schoenauer, Janne Cadamuro, Ulrike Kipman, Emma Stoellinger, Michael Lichtenauer, Vera Paar, Ludmilla Kedenko, Kathrin Guggenbichler, Bernhard Paulweber, Christian Pirich, Hermann Salmhofer

**Affiliations:** 1 Department of Nuclear Medicine and Endocrinology, Paracelsus Medical University, A-5020 Salzburg, Austria; 2 Department of Laboratory Medicine, Paracelsus Medical University, A-5020 Salzburg, Austria; 3 Independent Researcher, Landstraße 243, A-5424 Bad Vigaun, Austria; 4 Salzburg University of Applied Sciences, A-5412 Salzburg, Austria; 5 Department of Internal Medicine II, Paracelsus Medical University, A-5020 Salzburg, Austria; 6 Department of Internal Medicine I, Paracelsus Medical University, A-5020 Salzburg, Austria; 7 Outpatient Dialysis Unit Maxglan, Remisenweg 1, A-5020 Salzburg, Austria

**Keywords:** parathyroid hormone, secondary hyperparathyroidism, noxPTH, CKD, inflammation, oxidative stress, FGF23, VDBP

## Abstract

Intact parathyroid hormone (PTHi) plays a central role in the regulation of mineral and bone metabolism. Due to post-translational modifications of the hormone, the interpretation of elevated PTHi values is challenging and may benefit from an expanded analytical panel. Within this project, additional parameters of calcium–phosphate metabolism, such as non-oxidised parathyroid hormone (noxPTH), calcidiol, vitamin D binding protein (VDBP), and fibroblast growth factor 23 (FGF23) were evaluated in a control population of 177 individuals as well as 182 patients with renal, gastroenterological, and liver diseases. While PTHi and noxPTH levels were up to 10-fold higher in dialysis patients, the proportion of noxPTH on PTHi was significantly higher for all patient groups showing signs of inflammation. However, no strong confounders for PTHi could be identified. The correlation between CRP and the proportion of oxidised PTHi in total PTHi suggests an influence of inflammatory oxidative stress on the proportion of active noxPTH. Apart from the established role of vitamin D, the addition of noxPTH and its proportion of total PTHi in the assessment of unclear PTHi elevations seems reasonable, whereas there is no evidence for the standardised analysis of further parameters such as FGF23 and VDBP.

## 1. Introduction

Parathyroid hormone (PTHi) is one of the most important regulators of calcium and bone metabolism in the human body [1]. The large preprohormone with 115 amino acids is synthesised in the parathyroid gland and undergoes several modifications to obtain its activated form with 84 amino acids [2,3,4,5]. The hormone thus interacts with calcidiol (25 D3), vitamin D binding protein (VDBP) [6,7], and fibroblast growth factor 23 (FGF23) [8,9,10] in a precise manner to ensure calcium balance. In vivo, the hormone may be subject to several post-translational modifications that affect hormone function, some of which appear to be dependent on oxidative stress [11,12,13]. Indeed, it has been found that the methionine residues at positions 8 and 18 are available for oxidation processes [14]. As a result, some hormonal functions are reduced or lost [15]. In particular, oxidation at position 8 leads to a significant loss of function due to specific structural differences from the non-oxidised, active form. Therefore, the compensation of less active forms by a regulatory increase in hormone levels seems probable [16]. In addition, there seems to be evidence that hormone fragments—resulting from degradation [17,18] as well as from secretion by parathyroid master cells—may modulate the parathyroid hormone receptor 1 (PTH R1) [17]. These, as well as any posttranslationally modified forms of the hormone, do not underlie strict clearance processes [19,20]. This results in a 2–3-fold longer half-life of these PTH derivatives as compared to the intact non-oxidised hormone [21]. The slower rate of degradation and clearance causes accumulation of oxidised PTH and PTH-fragments predominantly in patients with chronic kidney disease (CKD) [22,23,24,25,26,27]. This might give rise to disturbed hormone-receptor interactions [23,28]. Further posttranslational modifications, such as phosphorylation of serine 17 [29,30], seem to occur rarely and with a strong association to patients with primary hyperparathyroidism and malignant tumours of the parathyroid gland. Yet to the best of our knowledge, this has not been analysed systematically, especially in renal patients [29,30,31]. Measurement of PTHi using a 3rd generation immunological assay detects all forms of post-translationally modified PTHi, but not hormone fragments (except for a cross-reactivity of fragment 7–84 [30]; but this specific fragment has not been verified in humans up to now [24,32,33]). Therefore, an increase in PTHi levels may seem disproportionate to biological needs, especially in patients with CKD and the associated oxidative stress. Consequently, this could be misleading in the diagnosis, treatment and monitoring of endocrine calcium metabolism [10,34]. A differentiation of quantities and species of PTH fragments, as well as non-oxidised and post-translationally modified PTHi, can be achieved by the use of liquid chromatography-mass spectrometry (LC-MS) [15]. However, due to restricted availability, this setting is not applicable for routine laboratory analysis. In contrast, the measurement of non-oxidised parathyroid hormone (noxPTH), after immunological extraction of the oxidised fraction within a rather simple preanalytical treatment, allows for the determination of the fraction of PTHi with full biological activity [12,13,35]. But, this test has not been established in routine diagnostics, as suggested by the sparsity of publications. This is possibly due to regulatory restrictions [36,37]. The clinical relevance of a noxPTH/PTHi ratio has, to the best of our knowledge, not yet been determined. Since levels of oxidative stress may largely change in the course of clinical diseases, this ratio may be subject to major intraindividual variations over time.

**Figure 1:**
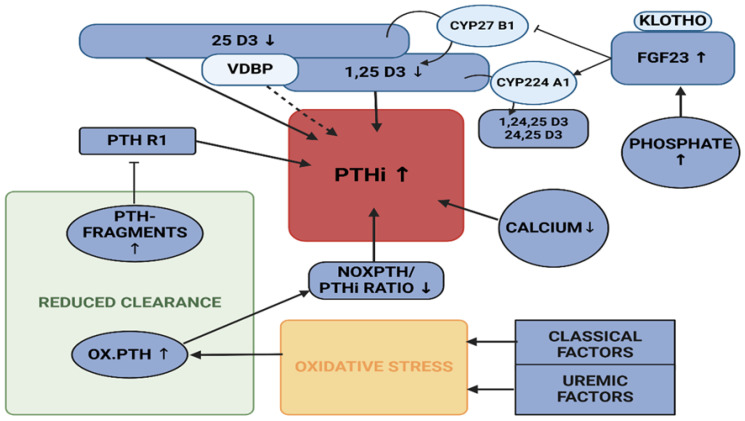
Driving forces in PTHi increase: Rising phosphate levels can lead to suppression of alpha 1 hydroxylase (CYP27 B1), as well as activation of 24 hydroxylase (CYP24 A1) via activation of FGF 23 in conjunction with Klotho, and thus to decline of 1,25-dihydroxy-vitamin D [44,45,46]. This is also a potential regulatory mechanism for the decrease of calcium and thus for the regulatory increase in PTHi [47,48]. Low 25 D3 levels also affect the same regulatory mechanism. Classical and uremic stress factors cause the oxidation of PTH molecules [42]. This reduction in effectiveness may induce an increase in total PTHi in order to maintain the hormone’s effect, resulting in a shift in the nox/PTHi ratio [12,13]. PTH fragments have a modulatory effect on PTH R1 and therefore contribute to an increase in PTHi [31]. In addition, a reduced clearance of fragments and oxidised PTH (oxPTH) leads to an amplification of this effect. The direct effect of VDBP on the system remains uncertain. Created in https://BioRender.com (accessed on 19 February 2025).

Further key players of calcium—phosphate metabolism, i.e., vitamin D, VDBP and FGF23, were included in this panel of analysis [6,35,38]. A decrease in calcidiol (25 D3), at levels below 30 ng/mL, may lead to a regulatory increase in PTH [39]. Calcitriol (1,25 D3), the corresponding active hormone, has a central role in calcium regulation; it promotes intestinal calcium and phosphate resorption to stabilise calcium blood levels and is necessary for bone mineralisation [39,40]. FGF23, the major phosphatonin, may also influence vitamin D levels. VDBP, in addition to its scavenger functions on cytoskeletal components, might also influence the regulation of vitamin D uptake and transport [7,41]. An overview of regulatory mechanisms is depicted in Figure 1.

The present study aimed to achieve a rational concept to improve the assessment of high PTHi levels. The majority of patients who would benefit from extended analysis of endocrinological parameters of calcium metabolism suffer from CKD. Renal failure is strongly associated with the effects of oxidative stress driven by conventional and uremic parameters [42,43]. Oxidation processes therefore influence analytical results in 3rd generation PTH assays [12,13,42,43]. However, patient groups affected by reduced vitamin D intake or inflammatory processes, such as those with chronic gastroenterological diseases, or patients with reduced protein biosynthesis, such as hepatological patients, may also be of interest in further extended analysis.

## 2. Results

Statistical considerations: The normal distribution tests revealed significant deviations from the Gaussian distribution pattern for all groups (0.001). Non-parametric group comparisons of all parameters using the Kruskal–Wallis test showed significant differences among groups (0.001). Within all patient groups, male sex predominated (>54.55%), whereas females predominated in the control cohort (63.69%). In addition, significant age differences between the control group and the dialysis group (0.001), as well as between the gastroenterology group and the dialysis group (0.001), could be detected. Furthermore, concerning all parameters investigated, no significant sex differences were found (0.001).

**Figure 2:**
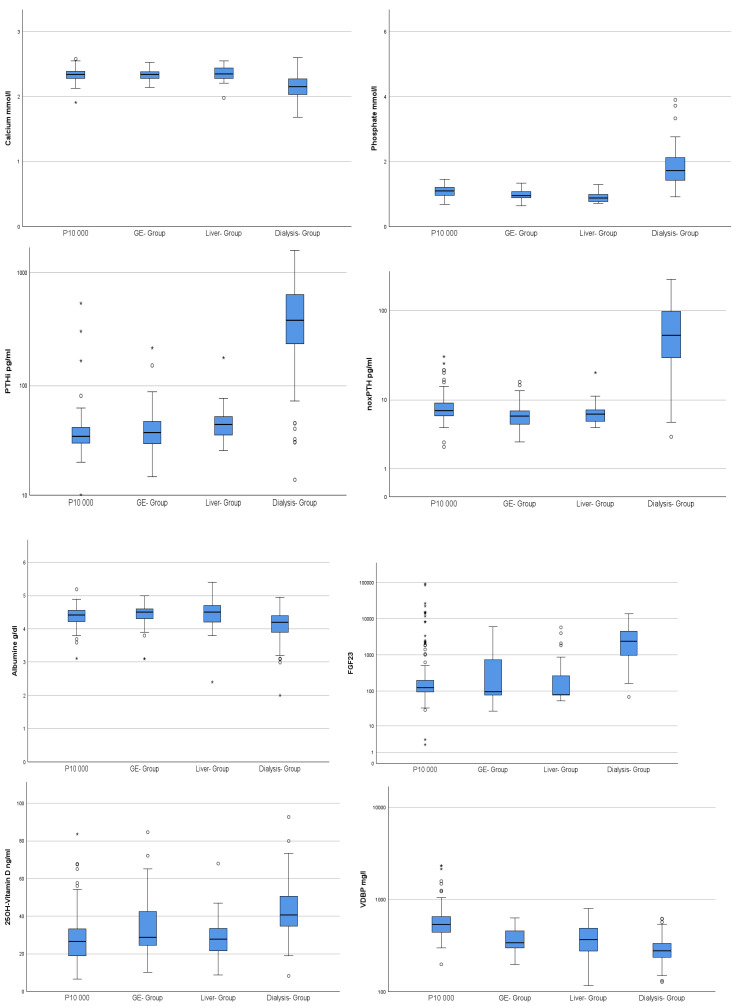
Differences between patients with chronic kidney disease and other patient cohorts. Demonstration of the differences between the dialysis group, the gastroenterological (GE-) and liver groups and controls. Significant differences for calcium, phosphate, PTHi, noxPTH, albumin, FGF 23, 25 D3 and VDBP, as well as for 25 D3 and VDBP, were found.

**Figure 3:**
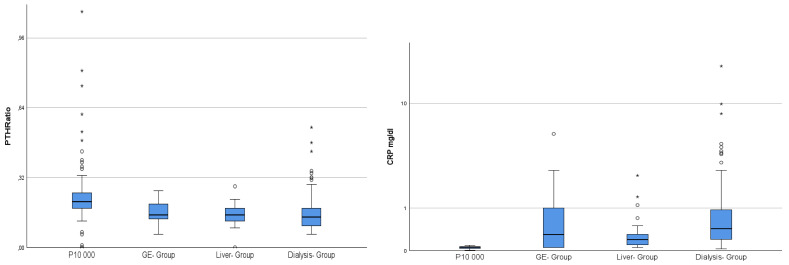
NoxPTH/PTHiRatio in relation to CRP as a surrogate marker for inflammation, presumably oxidative stress. The illustration shows the significantly higher proportion of the non-oxidised (0.001), active fraction of PTHi in the control population as compared to all patient groups examined, whereas CRP levels were significantly lower in the control population (0.001). Abbreviations: CRP, c-reactive protein; GE-group, gastro-enterological group; PTHRatio, noxPTH/PTHi Ratio.

Further analysis using the Mann–Whitney U test revealed the following relations: concerning phosphate, PTHi, noxPTH, 25 D3 and FGF23 (mean values are also given in Table 1), the dialysis group had significantly higher values than all other groups (0.001), whereas, for calcium and albumine (m = 4.39 g/dL), the values were significantly lower (0.001). In detail, PTHi (m = 478.8 pg/mL) and noxPTH levels (m = 68.99 pg/mL) were up to 10-fold higher in dialysis patients than in all other groups (PTHi < 49.48 pg/mL; noxPTH < 8.32 pg/mL) (Figure 2). VDBP was significantly higher in the control group (0.001) and significantly lower in the dialysis group (0.002), when compared with the—statistically equal—gastro-enterological and liver groups (0.366) (Figure 2). The parameters CRP and noxPTH/PTHi ratio showed significant differences between controls and all patient groups, with CRP being significantly lower (0.001) and noxPTH/PTHi Ratio being significantly higher (0.001) in the control group (Figure 3).

Multiple linear regression identified 25 D3 and FGF23 as weak confounders of PTHi with a predictive value of 9.1% (0.05) and coefficients of 5.53 (25 D3) and 0.04 (FGF23). noxPTH could not be included in the calculation due to collinearity. CRP and the noxPTH/PTHi ratio showed a moderate inverse correlation (R-0.465) with high significance (0.001).

## 3. Discussion

PTHi is widely used as a clinical laboratory parameter in various fields (such as osteology, nephrology, endocrinology), but has presented many analytical challenges. Different commercial assays give divergent results that remain unexplained [11,49,50,51], but induce additional effort in follow-up diagnostics. The role of post-translational modifications of PTHi, such as oxidation or phosphorylation, which might influence biological activity, is still a matter of debate [12,13,22]. Clinical interpretation of increased PTHi levels is difficult, since PTHi comprises active, as well as inactive fractions at an unknown and variable ratio. In this study, a panel of parameters related to bone and mineral metabolism was analysed in a control population (P 10,000) and several patient cohorts. PTHi and noxPTH were measured in parallel with vitamin D status, phosphate regulation, inflammatory activity, as well as basic electrolytes and renal function parameters. The driving mechanisms of PTH oxidation and the ratio of noxPTH to total PTHi have a major impact on PTHi blood levels and interpretation. In this project, we tried to identify factors that might influence the increase in PTHi and the rate of PTH oxidation under various clinical conditions.

Apart from dialysis patients, where secondary hyperparathyroidism and severe disturbances of mineral and bone metabolism are well established [8,10], we also included further patient groups with chronic inflammatory activity or presumed oxidative stress, such as gastroenterological and liver patients. In summary, we wanted to try and understand the conditions under oxidative stress, in normal renal function, as well as severe kidney disease.

The control population of P10,000 was selected based on the following criteria: normal weight (BMI < 25) non-smokers with normal electrolyte, renal, liver and inflammatory values, who did not develop any obvious diseases even during a 10-year follow-up [50]), included some individuals with unexpected high PTHi and FGF23 levels (Figure 2). Based on the long-term follow-up of the P10,000 cohort, tumour or severe renal-associated effects could be excluded to the best of our knowledge. Therefore, we decided to accept the relatively wide range of values in an inconspicuous, apparently healthy cohort.

**Figure 4:**
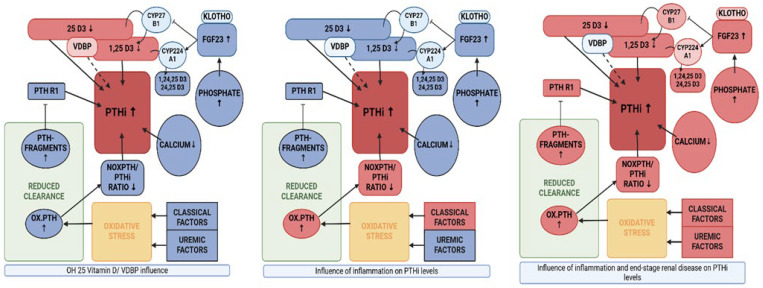
Illustration of several pathways to PTHi increase visualisation of the influences (highlighted in red) resulting from vitamin D deficiency [6], inflammation (as surrogate of oxidative stress; ref. [42]) and end-stage renal disease, which can result in a distinct increase in PTHi [47]. Created in https://BioRender.com (accessed on 19 February 2025).

In multiple regression analysis, only two weak confounders, calcidiol (25 D3) and FGF23, were identified with regard to total PTHi levels. This may be explained by effective vitamin D supplementation, which had been routinely implemented in the respective patient groups independently of this study. The slightly decreased range of 25 D3 in the control population [52] seems to result in a small but significant increase in VDBP when compared to all patient groups. In the dialysis group, significantly lower VDBP values were found at vitamin D sufficiency, when compared to all other groups (Figure 2). This suggests a self-regulatory mechanism. The predictive value of FGF23 for PTHi was low and presumably only driven by the phosphate imbalance of dialysis patients [47]. As our population was fairly replete with vitamin D, the confounding effects of vitamin D deficiency on mineral homeostasis and hormone regulation could be minimised. In conclusion, from our point of view, the analysis of FGF23 and VDBP does not add any diagnostic value, whereas early determination of vitamin D seems essential in stepwise diagnostics. The comparison of means of PTH values revealed significantly higher levels of PTHi and noxPTH in the dialysis cohort, which is similar to earlier studies [8,12,13,35]. In the present study, both parameters even showed up to 10-fold higher values than all other groups, whereas the calculated ratio of noxPTH/PTHi behaved differently. All patient groups revealed a significantly lower noxPTH/PTHi ratio when compared to the control population. This is comparable to the results of Seiler-Mussler et al. in 2015 [8] and might be due to increased oxidative stress in the respective diseased cohorts. Even more, this is in line with a moderate inverse correlation of CRP and noxPTH/PTHi ratio found for these groups. Dialysis patients, in addition to the aforementioned mechanism, have ancillary factors driving PTHi increase: Accumulation of PTH fragments and severe derangement of phosphate regulation need to be considered in the concept of uremic bone. In detail, several pathophysiologic patterns seem to emerge within our cohorts (Figure 4).

(i) Individuals without any apparent disease, recruited from the P10,000 cohort, presenting with increased VDBP, associated with rather low 25 D3 levels, but no statistical effect on PTHi or noxPTH.
(ii) Patients presenting with elevated CRP levels with a reduced proportion of noxPTH, while renal and mineral metabolism seem undisturbed. This may be due to an inflammatory process and, presumably, oxidative stress.
(iii) Patients, whose end stage renal disease is likely to upregulate the level of PTHi and noxPTH via several mechanisms: (a) Impaired clearance of non-functional or only partially functional, oxidised PTH [20], (b) decreased clearance of receptor modulating PTH fragments [21,23,24,53], (c) The regulatory cascade of renal function decline, starting with deteriorating phosphate clearance, ensuing increase in FGF23 levels, decrease of 1,25 D3 and hypocalcaemia, resulting in secondary renal hyperparathyroidism [47].

The inclusion of gastroenterological and liver patient cohorts in our study allows evaluation of inflammatory and oxidative stress conditions, yet without the additional features of end-stage renal disease. This may indicate that oxidative stress can lead to a significant downward shift in the proportion of active noxPTH, causing a regulatory increase in PTHi. Interestingly, a similar shift in the ratio was found in all patient groups. At the same time, mechanisms specific to end-stage renal disease, such as the phosphate driven, FGF 23/klotho induced inhibition of 25 D3 1-alpha hydroxylation, or the accumulation of inactive PTH fractions and fragments, have a high force on the increment of PTH, resulting in up to tenfold increased levels of both noxPTH and PTHi [23,24,53]. Of note, even the bioactivity of fully functional noxPTH may be compromised by receptor interaction of fragments [31]. This novel aspect would require complex analysis and should be the subject of future research.

Since PTHi comprises active (non-oxidised) and inactive (oxidised) fractions to variable degrees, clinical interpretations of increased PTHi blood levels are complex. Vitamin D deficiency must first be ruled out or corrected, and renal function must be evaluated in parallel. Conclusively, determination of nox PTH and calculation of the noxPTH/PTHi ratio may facilitate further interpretation by indicating putative influences of inflammatory activity, oxidative stress and the uremic milieu.

## 4. Materials and Methods

**Figure 5:**
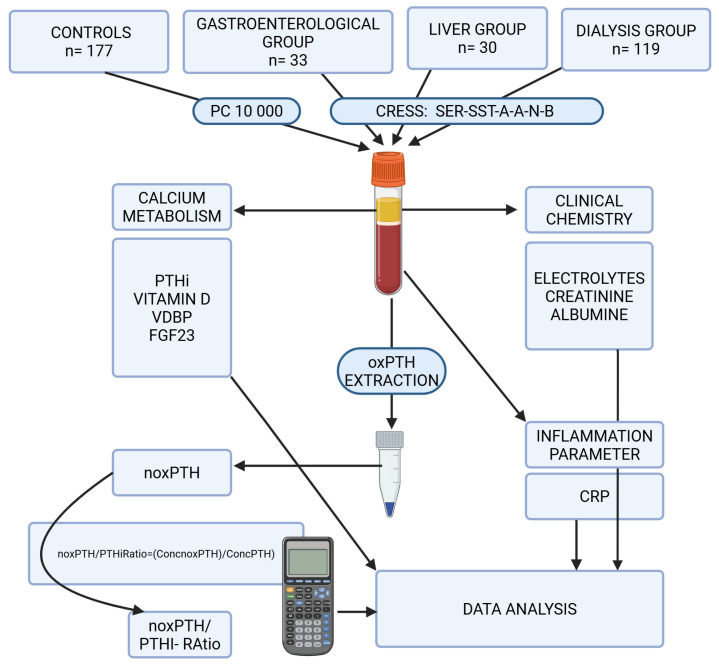
Visualisation of the study design. Samples were either obtained from residual sample volumes, derived from routine laboratory examinations, and collected according to SER-SST-A-A-N-B [54] or from the Paracelsus 10,000 cohort [55]. All extraction and analysis procedures were performed within 24 h and after a maximum of one thawing cycle. The percentage of inactive or partially active oxidised PTHi was calculated, and data analysis was performed using SPSS 29.0.2.0 ()20) (IBM, 1 New Orchard Road, Armonk, New York 10504-1722, NY, USA). Created in https://BioRender.com (accessed on 19 February 2025). Laboratory facilities: Sample selection, preanalytical treatments and analysis of PTHi, noxPTH, 25 D3 and VDBP were performed in the endocrinological laboratory of the Paracelsus Medical University Salzburg, between February 2024 and June 2024. At the same time, analysis of electrolytes, albumin, creatinine and CRP was performed at the Department of Laboratory Medicine, University Hospital Salzburg. Both laboratories are certified according to ISO 9001: 2015 and work according to the accreditation standard for medical laboratories ISO 15189: 2022 [56,57]. The analysis of FGF23 was performed in the Laboratory of Internal Medicine II, which works according to the accreditation standard for medical laboratories ISO 15189: 2022 [57].

Study population: The study population included 177 (m = 65/f = 112) individuals from the Paracelsus 10,000 cohort as a control group, i.e., non-smokers of normal weight (BMI < 25) with normal electrolyte, kidney, liver and inflammatory values, who did not develop any obvious diseases during the 10-year follow-up [49]. In addition, several clinical cohorts, including 119 (m = 81/f = 38) dialysis patients and 33 (m = 18/f = 15) individuals from a gastroenterological outpatient clinic (predominantly with chronic inflammatory bowel diseases). A further 30 (m = 19/f = 11) individuals were recruited from a liver outpatient clinic, with pathologies including various stages of non-alcoholic and alcoholic liver diseases and cirrhosis. Samples were collected as residual material of routine diagnostic procedures (Figure 5). For the clinical cohorts, BMI was not available due to the limited access to patient information during the selection process.

Study design: The recruitment of patient groups and controls, as well as sample and data workflow, are summarised in Figure 5.

**Figure 6:**
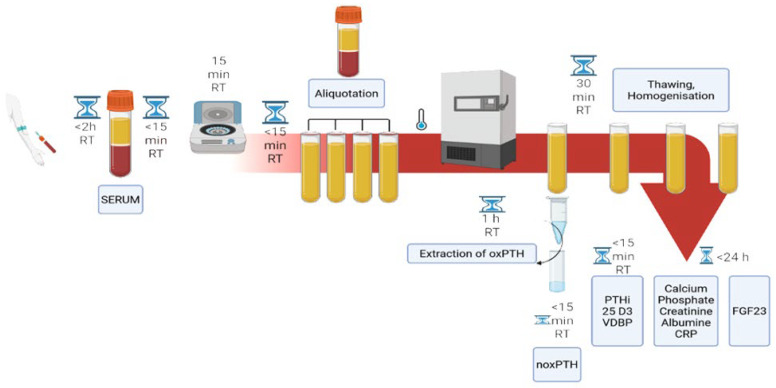
Pre-analytical procedures, storage times and temperatures during this study. Created in https://BioRender.com (accessed on 19 February 2025).

Sample acquisition and sample processing: Material for analysis was collected from residual samples of routine diagnosis in dialysis, gastrointestinal and hepatic patients (Figure 5). As this was a quality assessment study and no additional specimens were collected, it was essential to adapt the study design to real-world preanalytical conditions in the clinic, as required by the Institutional Review Board. Accordingly, serum (as suggested by all manufacturers’ specifications) was used for analysis according to routine procedures. Blood was collected under standardised conditions [54] using Greiner Safety Blood Collection and BD Vacutainer^tm^ SST TM II Advance tubes with gel separator (Greiner Bio-One GmbH, Bad Haller Str. 32, A-4550 Kremsmünster, Austria). After blood collection, the tubes were left at room temperature (18–28 °C) for 15 min to allow for complete clotting. Samples were then centrifuged at 3000× *g* at room temperature, aliquoted and frozen within two hours of collection (including transport time for liver and gastroenterological patients). The freezing period for the gastroenterological group, liver group and dialysis group did not exceed 2 months at −20 degrees Celsius. Sample processing is represented by CRESS: SER-SST-A-A-N-B [49] (Figure 2). The Paracelsus 10,000 cohort served as a control group. Blood was collected after a 10 h fast using Vacutainer blood collection systems (Greiner Bio-One™) [50] (Figure 5). After blood collection, these tubes were also allowed to clot for 15 min at room temperature (18–28 °C), centrifuged at 3000× *g* at room temperature and stored at −80 °C within two hours at room temperature for a maximum of five years until further analysis. All samples were thawed once at room temperature (30 min), mixed, inverted and added to the appropriate assay or immunoassay for oxPTH within 15 min. For noxPTH measurement, analysis was performed within 15 min of sample preparation using Paratrin Prolife (Figure 6). No haemolytic, icteric or lipemic samples were used in this study.

Within the scope of the study design, particular attention was paid to the storage stability of the samples in the determination of PTH and FGF 23 and therefore, with respect to manufacturer’s specifications and the recent literature, storage at room temperature did never exceed two hours, the samples were not thawed more than once and long term storage at −20 degrees did not exceed 2 months [58,59].

Sample analysis: Analysis of PTHi was performed using the Roche Elecsys PTHi stat (Roche Diagnostics, Forrenstrasse 2, CH-6343 Risch-Rotkreuz, Basel, Switzerland) third-generation chemoluminescence assay. For analysis of noxPTH, the oxidised hormone fraction was removed from the samples by incubation of 300 µL serum samples on Paratrin Prolife^®^ columns (A1112, ImmunDiagnostik AG, Stubenwald-Allee 8a, D-64625 Bensheim, Germany) and the remaining noxPTH was analysed, again using the Cobas System. Quality assurance for the Cobas System was performed daily at three levels using Cobas Varia QC material. Alinity 25-OH-Vitamin D (Abbott Laboratories, 100 Abbott Park Road, Abbott Park, IL 60064 Chicago, IL, USA) one-step chemoluminescent microparticle assay, was used for the measurement of 25 D3. Internal quality control was performed using Technopath quality control material Multichem IA plus. After 40.000-fold pre-dilution of the samples, VDBP was analysed using VDBP Elisa (K2314 Immundiagnostik, Bensheim, Germany), by measurement with the TECAN Sunrise ELISA-Reader and calculation using the TECAN Magellan 7.3 Software (Tecan, Seestrasse 103, CH-8708 Männedorf, Switzerland). Serum FGF23 was measured by enzyme-linked immunosorbent assay from R&D Systems (DY2604-05, 19 Barton Lane, Abingdon Science Park, Abingdon, OX14 3N, UK). The results were automatically calculated using the microplate manager software MPM6 version 6.3 (Bio-Rad, Kapellenstraße 12, 85622 Feldkirchen, Germany). Serum electrolytes, albumin, creatinine and CRP were measured using a COBAS 8100 instrument (Roche Diagnostics, Basel, Switzerland), whereby quality controls were measured twice daily at different concentrations. The methods, production and performance parameters of the tests are listed in Table 2.

**Calculated parameters**: The noxPTH/PTHi ratio was automatically calculated, using Excel 2016 (Microsoft 365; One Microsoft Way, Redmond, WA 98052-6399, USA), by means of the following function and represents the proportion of non-oxidised PTH to total PTHi.

noxPTH/PTHiRatio = (Conc_noxPTH)/Conc_PTHi)

**Limitations:** Due to the setting as a quality management project, the patient numbers in the gastroenterological and liver cohorts were limited. It was therefore not possible to achieve the desirable balance in terms of age and sex. There was heterogeneity in vitamin D supplementation, which may have influenced the data of this work. Due to the small residual sample volumes available, it was not possible to analyse further parameters of oxidative stress apart from the inflammatory parameter CRP.

## Figures and Tables

**Table 1:** Statistical indicators of all measured parameters for each patient group. Abbreviations: PTHi, intact parathyroid hormone; noxPTH, non-oxidised parathyroid hormone; 25 D3, 25-hydroxy-vitamin-D3; VDBP, vitamin D binding protein; FGF23, fibroblast growth factor 23.

	P10,000 *n* = 177		GE-Group *n* = 33		Liver-Group *n* = 29		Dialysis *n* = 119	
	Mean	SD	Mean	SD	Mean	SD	Mean	SD
Age years	53.95	2.64	48.9	16.52	56.37	16.78	64.13	17.36
Calcium mmol/L	2.33	0.09	2.34	0.09	2.35	0.12	2.15	0.18
Phosphate mmol/L	1.09	0.16	0.97	0.17	0.91	0.17	1.85	0.72
Creatinine mg/dL	0.80	0.13	1.12	0.96	0.98	0.29	8.71	2.88
CRP mg/dL	0.06	0.02	0.69	1.13	0.35	0.50	1.06	2.21
Albumine g/dL	4.39	0.27	4.37	0.45	4.41	0.54	4.12	0.45
PTHi pg/mL	41.93	44.72	47.70	38.91	49.48	27.41	478.80	343.35
noxPTH pg/mL	8.32	3.64	6.75	3.00	7.14	3.03	68.99	51.82
noxPTH/PTHi Ratio	0.23	0.12	0.16	0.04	0.15	0.05	0.16	0.08
25 D3 ng/mL	26.92	12.76	34.45	16.26	28.70	12.56	42.66	12.61
VDBP ng/mL	1144.94	5111.95	385.60	120.70	405.16	168.99	294.62	95.77
FGF23 pg/mL	2151.06	10,156.76	898.77	1524.48	642.76	1354.57	3268.43	3053.23

**Table 2:** Presentation of analytical performance parameters which provides information on the sensitivity, likely cross-reactivity, and coefficient of variation in the analytical method used, according to the manufacturer’s specifications. The coefficient of variation for the measurement of PTHi after extraction of oxidised PTH using Paratrin Prolife was taken from the work of Tepel et al. [35].

Analysis	Manufacturer	Method	Sensitivity	Specificity/Cross Reactivity	CV%
Albumine	Roche	Extinction	2 g/L	no interference	<0.8
Calcium	Roche	Extinction	0.2 mmol/L	no interference	<1.5
FGF 23	BioTechne	ELISA	78.1 pg/mL	no cross-reactivity	n.d.
CRP	Roche	Extinction	na	no interference	<3.3
Creatinine	Roche	Extinction	0.2 mg/dL	no interference	1.4
Phosphate	Roche	Extinction	0.1 mmol/L	no interference	<1.4
PTHi	Roche	ECLIA	1.2 pg/mL	cross-reactivity of 93% with the PTH fragment 7-84	<3.4
Paratrin Prolife	ImmunDiagnostik	Immunologic	n.d.	cross-reactivity of 93% with the PTH fragment 7-84	<5.8% including subsequent analysis by PTHi/Roche [35]
VDBP	ImmunDiagnostik	ELISA	0.944 ng/mL	no cross-reactivity	<13.9
25 D3	Abbott	CMIA	3.5 ng/mL	cross reactivity of >80.5% with OH D2 >70.4% with 24.25 OH D3 and 24.25 D2	<8.3

## Data Availability

The datasets presented in this article are not readily available because the data are part of a further ongoing study. Generally, original patient data are not publicly available, being protected by the EU General Data Protection Regulation 2016/679 and specific Austrian health data protection laws. Requests to access the datasets should be directed to u.huber-schoenauer@salk.at.

## Abbreviations

The following abbreviations are used in this manuscript:

25 D3	25-hydroxy-vitamin D3
1,25 D3	1,25-dihydroxy-vitamin D3
CKD	chronic kidney disease
CMIA	chemoluminescent microparticle immunoassay
CV	variation coefficient
conc.	concentration
CRP	C-reactive protein
ECLIA	electrochemiluminescence immunoassay
ELISA	enzyme linked immunosorbant assay
FGF23	fibroblast growth factor 23
LC-MS	liquid chromatography-mass spectrometry
noxPTH	non-oxidised parathyroid hormone
noxPTH/PTHi Ratio	non-oxidised parathyroid hormone/intact parathyroid hormone ratio
oxPTH	oxidised parathyroid hormone
PTH R1	parathyroid hormone receptor 1
VDBP	vitamin D binding protein
